# CD109, a master regulator of inflammatory responses

**DOI:** 10.3389/fimmu.2024.1505008

**Published:** 2025-02-07

**Authors:** Adel Batal, Setareh Garousi, Kenneth W. Finnson, Anie Philip

**Affiliations:** ^1^ Division of Plastic Surgery, Department of Surgery, McGill University, Montreal, QC, Canada; ^2^ Surgical and Interventional Sciences Program, The Research Institute of the McGill University Health Centre, Montreal, QC, Canada

**Keywords:** CD109, inflammatory response, TGF-β signaling, NF-κB signaling, pathway crosstalk

## Abstract

Inflammation is a complex response to harmful stimuli, crucial for immunity, and linked to chronic diseases and cancer, with TGF-β and NF-κB pathways as key regulators. CD109 is a glycosylphosphatidylinositol (GPI)-anchored protein, that our group has originally identified as a TGF-β co-receptor and inhibitor of TGF-β signaling. CD109 modulates TGF-β and NF-κB pathways, to influence immune responses and inflammation. CD109’s multifaceted role in inflammation spans various tissue types, including the skin, lung, bone and bone-related tissues, and various types of cancers. CD109 exerts its effects by modulating processes such as cytokine secretion, immune cell recruitment, macrophage polarization, T helper cell function and cancer cell phenotype and function. Here, we review CD109’s regulatory functions in inflammatory responses in these various tissues and cell types. Exploration of CD109’s mechanisms of action will enhance our understanding of its contributions to disease pathology and its potential for therapeutic applications.

## 1 Introduction

Inflammation is the body’s alarm system, triggered by injuries or infections. This response manifests through symptoms like redness, swelling, pain, and fever, all aimed at signaling that something is wrong (1). It is a protective mechanism for fighting threats and facilitating healing. However, inflammation can sometimes become problematic, turning into a chronic state that contributes to diseases such as cancer, arthritis, and fibrosis. CD109, while not yet widely recognized as a regulator of the immune system, has recently emerged as a pivotal player in the inflammatory landscape.

CD109 is a glycosylphosphatidylinositol (GPI)-anchored protein, first identified as a marker for activated T cells and platelets (2) and was later shown to be preferentially expressed on hematopoietic stem and progenitor cell subsets (3). Early studies also demonstrated that the Gov alloantigen involved in immune reactions to platelet transfusions is localized to CD109 (4). The first cloning of CD109 was reported in 2002 by Lin et al. (5), who identified it as a gene encoding a 1445 amino acid (aa) protein of approximately 162 kDa with an N-terminal signal sequence and a C-terminal GPI anchor cleavage-addition site. They demonstrated that CD109 is a novel member of the α2 macroglobulin (α2M)/C3, C4, C5 family of thioester-containing proteins with a putative bait region, a furin cleavage and a hexapeptide critical for thioester reactivity and suggested that it may have potent immune regulatory functions (5). Following this, based on a phylogenetic analysis of human CD109 with other human homologs as well as orthologs from other mammalian species, C. elegans (ZK337.1) and E. coli homologs, Solomon et al. proposed that CD109 represents a novel and independent branch of the a2-M/complement gene family and may be its oldest member (6). Although the specific function of CD109 remained unknown with no information on a ligand or an interacting receptor for CD109 (6), together the above two studies provided the foundational understanding of CD109’s structure and possibility to study its potential role in cellular function and immune regulation.

Our group was the first to report a specific cellular function for CD109 by showing that CD109 binds to Transforming Growth Factor-β (TGF-β) with high affinity and that it is a TGF-β coreceptor forming a component of the TGF-β signaling complex (7). In the above study, we demonstrated that CD109 represents r150, a molecule we have previously characterized as a TGF-β binding protein (8–10). Furthermore, the study also showed that CD109 is a potent inhibitor of TGF-β signaling and responses such as extracellular matrix (ECM) synthesis. In addition, CD109 directly interacts with TGF-β signaling receptors and modulates their signaling activity (8–10). The finding that CD109 is a negative regulator of TGF-β signaling is of particular significance, given that TGF-β is a central mediator of inflammation with a broad spectrum of immune regulatory effects.

In later studies using mice overexpressing CD109 in the epidermis, our group has showed that CD109 overexpression leads to markedly reduced inflammatory responses including macrophage and neutrophil recruitment, granulation tissue formation during wound healing, when compared to wild-type littermate controls, while wound closure remains unaffected (11). Additionally, the CD109-mediated decrease in inflammatory responses in the CD109 overexpressing mice was associated with decreased TGF-β/Smad2/3 signaling (11) and Nuclear Factor kappa B (NF-κB) pathway inhibition (12, 13) as well as amelioration of bleomycin-induced skin fibrosis (14). In contrast, CD109 knockout mice display epidermal hyperplasia, impaired hair growth, and heightened immune cell infiltration (15) while exhibiting enhanced TGF-β/Smad2/3 signaling and increased skin fibrosis (16). The above gain and loss of function studies underscore CD109’s critical role in regulating inflammatory responses. Furthermore, as CD109 appears to exert its influence by modulating the two key inflammatory signaling pathways, the TGF-β/Smad2/3 and the NF-κB pathways, CD109 may play a crucial in balancing inflammation by modulating these pathways. Thus, CD109’s role in this balance could determine whether inflammation remains protective or becomes harmful. A well-regulated response aids healing, while an uncontrolled one can lead to chronic diseases.

Although CD109 was initially thought to have a limited tissue distribution, it was later reported to be expressed widely in different cell types including keratinocytes, fibroblasts, chondrocytes, and the testis (7). Elevated CD109 expression has been linked to various conditions, including skin diseases, platelet disorders, and cancers, all of which have a significant immune component. CD109 levels are increased in various cancers. As mentioned earlier, the biallelic platelet-specific Gov antigen system, also known as HPA-15, which is associated with platelet disorders such as platelet transfusion refractoriness, neonatal alloimmune thrombocytopenia, and posttransfusion purpura, is localized to CD109 (17, 18). The Gov alleles differ by an A to C single nucleotide polymorphism (SNP) at position 2108 of the coding region, leading to a Tyr/Ser substitution at amino acid 703 of CD109. This SNP in CD109 expressed on activated platelets is strongly associated with immune reactions leading to platelet destruction, alloimmune thrombocytopenia and serious clotting (17). Elevated expression of CD109 has been implicated in the progression of cancers including squamous cell carcinoma, glioblastoma, prostate carcinoma, basal-like breast carcinoma, and certain adenocarcinomas and sarcomas (19–24). While its presence in activated T-cells suggested a role in immune responses (2), its overexpression in tumors suggests involvement in cancer progression (25). The skin disorders in which CD109 has been shown to play a key role include lung fibrosis, scleroderma, psoriasis, and keloids (26–29). Aberrant TGF-β and NF-κB signaling are known to aggravate tissue damage in both inflammatory and infectious diseases by enhancing inflammation, increasing susceptibility to pathogen infections, and disrupting normal tissue remodeling processes (30). CD109, as the major endogenous negative regulator of those signaling pathways, is likely to play a pivotal role in those pathophysiological processes.

In this review, we will explore the multifaceted role of CD109 in regulating inflammation and immune responses across various cell types, with a focus on its interactions with the key inflammatory signaling pathways.

## 2 Role of CD109 in regulating TGF-β and NF-κB signaling and crosstalk

TGF-β is a pleiotropic cytokine that plays fundamental roles in development and homeostasis by strongly regulating immune responses and other processes including cellular proliferation, differentiation, and ECM production (31). The three mammalian isoforms of TGF-β (TGF-β1, -β2, and -β3) share high (75%) sequence and structural similarity, but perform distinct functions *in vivo* (31). These isoforms are often described as having overlapping functions *in vitro* (32). However, *in vivo* studies in knockout mice show non-redundant phenotypes (33, 34), with TGF-β1 having a specific role in inflammation. TGF-β1 enhances the inflammatory response by upregulating expression of pro-inflammatory cytokines IL-1, IL-8, TNF-α, PDGF, FGF-2, and MCP-1 (35, 36).

We have previously reported that CD109 exhibits isoform specificity in binding to TGF-β (7) with high affinity for the TGF-β1 isoform, lower affinity for TGF-β3, and virtually little affinity for TGF-β2, with CD109 showing the most potent effect in inhibiting TGF-β1-mediated responses (9, 10). In addition, our findings also revealed that endogenous CD109 can be released from the cell surface and that the released/soluble CD109 also bind to TGF-β with high affinity, neutralizing TGF-β activity and TGF-β-mediated responses (7, 10, 37). Thus, CD109 in both its membrane-anchored form and soluble form inhibits TGF-β responses. Notably, CD109 stands out as the most potent negative regulator of canonical TGF-β signaling and responses.

As indicated above, CD109 represents a TGF-β co-receptor, regulating TGF-β signaling as a component of the TGF-β receptor complex (7, 8, 10, 29, 37), with CD109 interacting with TGF-β signaling receptors both in the presence and absence of the TGF-β ligand (7). The mechanism by which membrane-anchored CD109 inhibits TGF-β signaling involves CD109 promoting the localization of TGF-β receptors into the caveolar compartment in a TGF-β-dependent manner and enhancing the internalization and Smad7 and Smurf-2-mediated degradation of TGF-β receptors, as we demonstrated previously (38, 39).

The TGF-β signaling pathway is mediated by a pair of transmembrane serine/threonine kinase receptors: type I receptor (TβRI), also called activin receptor-like kinase-5 (ALK5), and type II receptor (TβRII) (31). In the canonical TGF-β pathway, TGF-β binding to TβRII results in TβRI phosphorylation leading to the activation of the receptor-regulated Smads (rSmads) Smad2 and Smad3 (40). Activated Smad2/3 proteins form a complex with Smad4, enter the nucleus and regulate gene expression in concert with transcriptional co-activators and co-repressors (31, 40). The inhibitory Smad (I-Smad), Smad7 is a nuclear protein which acts as a key negative regulator of the TGF-β signaling pathway. Smad7 binds to Smurf2, an E3 ubiquitin ligase, in the nucleus, translocating it to the cytoplasm in response to TGF-β, resulting in the recruitment of the Smad7/Smurf2 to the activated TGF-β receptor complex, leading to TGF-β receptor ubiquitination, followed by proteasomal/lysosomal degradation (31).

In addition to the canonical TGF-β signaling pathway, there are multiple non-canonical pathways that TGF-β can activate which are highly context dependent. Activation of non-canonical TGF-β pathways can be influenced by various factors, including cell type, microenvironment, presence of specific co-factors or interacting proteins, and the overall cellular signaling landscape (41). This context-dependent choice allows cells to fine-tune their responses to TGF-β signaling based on the specific requirements of the physiological or pathological conditions. Some key non-canonical pathways include ALK1/Smad1/5 (42), MAPK (ERK, JNK/p38) (41), PI3K/Akt (43), and NF-κB pathways, among others. The NF-κB pathway is the major TGF-β non-canonical pathway that plays a crucial role in inflammation and immune responses (44–48), with the TGF-β/Smad2/3 and NF-κB pathways reciprocally regulating each other’s activity via crosstalk (49).

TGF-β activates NF-κB signaling through a sequential regulation of TGF-β-activated kinase 1 (TAK1) and IkB kinases (IKKs) (which involves TAK1 binding to TANK-binding Kinase 1), leading to phosphorylation of Inhibitor of NF-κB alpha (IκBα), nuclear translocation and phosphorylation of NF-κB subunit p65 and activation of NF-κB downstream targets, driving inflammatory responses (44, 50).

NF-κB is a critical transcription factor that regulates the immune responses and inflammation, in addition to cell survival and proliferation. NF-κB mediates pro-inflammatory gene induction in innate and adaptive immune cells (51). Furthermore, NF-κB signaling regulates the NLRP3 inflammasome pathway, essential for the induction of inflammation in response to infectious pathogens (52).The NF-κB canonical pathway is downstream to various receptors: Toll-Like Receptors (TLRs), Il-1/IL-8 receptors, TNF receptor superfamily, and B (BCR) and T (TCR) cell receptors (53). Each receptor employs different signaling components and adaptor proteins to induce NF-κB activation, with MyD88 universally recruited to all receptor complexes except TLR3 (54). The NF-κB/REL family is composed of five members: p65 (RelA), c-Rel, RelB, p50 (NF-κB1; and its precursor p105), and p52 (NF-κB2; and its precursor p100) (54). The RelA and p50 heterodimers are responsible for the canonical transcription of target genes (54). The RelB and p52 heterodimers are involved in the non-canonical gene transcription (54). Before activation, RelA and p50 are bound to the IκB proteins in the cytoplasm (54). To activate NF-κB, IKKs need to phosphorylate IκBα, which leads to K48-linked ubiquitination of IκB (54). The polyubiquitinated IκB will then undergo proteasome-mediated degradation (54). The degradation of IκB is the main event in the canonical NF-κB pathway since it liberates the RelA/p50 heterodimer for activation and translocation into the nucleus (54). Once in the nucleus, the NF-κB heterodimer will regulate the transcription of various cytokines (TNF-α, IL-1, IL-6), chemokines (RANTES, CXCL-10, MCP-1), adhesion molecules (ICAM-1, VCAM-1), and anti-apoptotic factors (Bcl-2) (55). Canonical NF-κB regulates the transcription and maturation of IL-1β by first inducing pro-IL-1β transcription, and then cleavage by caspase-1 (56).

Unlike the canonical NF-κB pathway, the non-canonical pathway specifically responds to a distinct set of stimuli. These include ligands for certain members of the TNF receptor superfamily, such as LTβR, BAFFR, CD40, and RANK (54). In this pathway, the RelB/p52 heterodimer is the main NF-κB transcription factor involved (54). The main event in this pathway is the processing of the p100 precursor into p52, via phosphorylation by NF-κB-inducing kinase (NIK) and IKKα (57).

The crosstalk between TGF-β/Samd2/3 and NF-κB signaling pathways significantly influences immune and inflammatory responses through both synergistic and antagonistic mechanisms (44). NF-κB signaling can inhibit TGF-β signaling to support T cell activation by activating Caspase recruitment domain-containing membrane-associated guanylate kinase protein-1 (CARMA1) in response to TCR engagement (58). This mechanism allows NF-κB and CARMA1 to overcome the suppressive effects of TGF-β signaling, thus promoting T cell activation (58). Additionally, Smad7, induced by NF-κB, forms complexes with TGF-β activated kinase binding proteins 2 and 3 (TAB2 and TAB3), inhibiting TNF-α signaling and thereby reducing NF-κB activity (59). The p65 (RelA) subunit inhibits TGF-β/Smad signaling by inducing Smad7 synthesis, thereby suppressing TGF-β-induced phosphorylation, nuclear translocation, and DNA binding of Smad complexes in response to TNF-α and other pro-inflammatory stimuli (60). This highlights the role of Smad7 as a mediator that limits inflammatory responses by antagonizing NF-κB signaling. Moreover, TGF-β induces the ubiquitination and degradation of MyD88 through Smad6 and Smurf proteins, negatively regulating pro-inflammatory signaling (61). Conversely, there are synergistic aspects of the crosstalk between NF-κB and TGF-β pathways, particularly in enhancing inflammatory responses. In glioblastoma, for instance, NF-κB activation upregulates TGF-β via miR-148a or miR-182, leading to the hyperactivation of both pathways and thereby enhancing inflammation and immune response dysregulation (62, 63). Recent clinical studies have increasingly focused on the TGF-β and NF-κB signaling pathways, highlighting their complex potential interplay in various diseases. Isochlorogenic acid A reduces lead-induced liver inflammation and fibrosis by inhibiting the TGF-β1/Smad2/3 and NF-κB signaling pathways, leading to decreased expression of inflammatory cytokines and collagen deposition (64). Palmitic acid increases kidney injury in diabetic kidney disease by promoting inflammation and fibrosis through the TLR4/MyD88/NF-κB and TGF-β/Smad signaling pathways (65). Particulate matter exposure worsens airway inflammation and pulmonary fibrosis via TXNIP/NF-κB and SIRT1/TGF-β/Smad3 pathways, causing increased inflammation and impaired lung function (66). Parthenolide, a compound that treats peritoneal fibrosis, reduces levels of inflammatory cytokines, decreases TGF-β1 expression and the phosphorylation of IκBα and p65, thereby inhibiting the TGF-β/Smad/NF-κB signaling axis and demonstrating Parthenolide’s potential as a therapeutic agent for inflammation-induced fibrosis (67).While the above studies independently highlight the roles of TGF-β and NF-κB signaling pathways in inflammation and fibrosis, they do not explicitly address any direct link or potential crosstalk between them.

**Figure 1:**
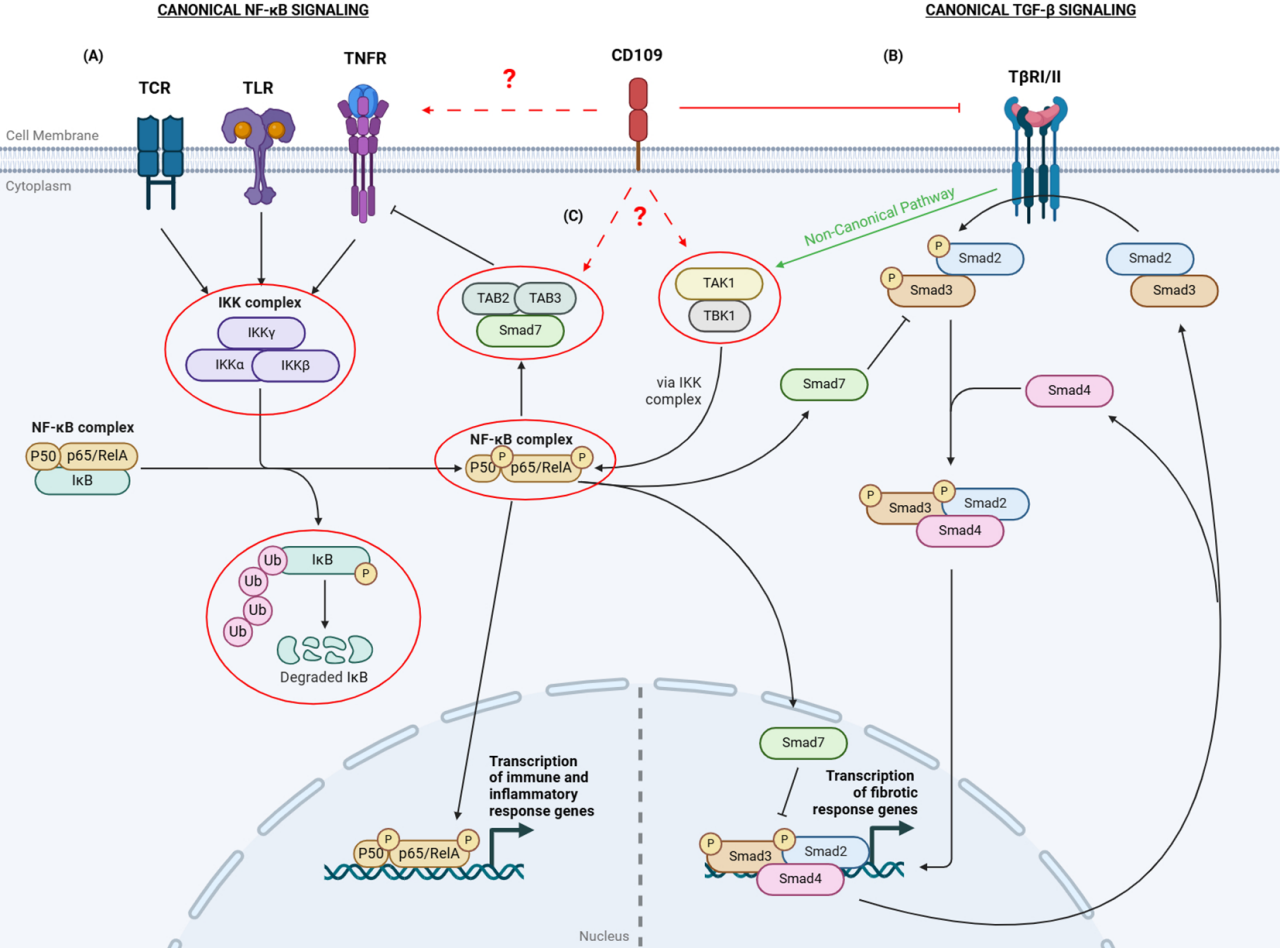
Overview of CD109-mediated regulation of TGF-β and NF-κB signaling. **(A)** The canonical NF-κB pathway is activated by receptors such as TCRs, TLRs, and TNFRs. Ligand binding to these receptors activates IKKs to phosphorylate IκB. Phosphorylated IκB dissociates from the NF-κB complex (p50 and p65/RelA), gets polyubiquitinated, and is degraded. The p50/p65 complex then translocates to the nucleus to activate immune response genes. **(B)** In the canonical TGF-β signaling, when TGF-β binds to the TGF-β Receptor I and II complex, it leads to the phosphorylation of Smad2/3. The activated Smad2/3 then form a complex with Smad4 and translocate to the nucleus to regulate the transcription of target genes. Smad7 can inhibit TGF-β signaling by either preventing Smad2/3 phosphorylation or by inhibiting the transcriptional activity of the nuclear Smad complex. **(C)** The TGF-β and NF-κB canonical pathways crosstalk with each other through various mechanisms.TGF-β can activate TAK1 and TBK1, which in turn activate the IKK complex and promote NF-κB signaling.The p65 subunit can enhance Smad7 expression, inhibiting TGF-β signaling. Additionally, p65 can enable Smad7 to bind to TAB2/3, inhibiting TNF-α receptor activation, reducing inflammatory responses. CD109 inhibits canonical TGF-β signaling. The specific role of CD109 in the canonical NF-κB pathway and its crosstalk with the TGF-β pathway remains unclear. The red circles and dashed arrows with a question mark indicate targets potentially regulated by CD109. TCR: T Cell Receptor; TLR: Toll-Like Receptor; TNFR: TNF-α Receptor; TβRI/II: TGF-β Receptor I/II; IκB: Inhibitor of NF-κB; IKK: IκB kinases; TAB2/3: TGF-β Activated Kinase Binding Proteins 2 and 3; TAK1: TGF-β-Activated Kinase 1; TBK: TANK-binding Kinase. *Created with* *BioRender.com* .

The relationship between TGF-β and NF-κB signaling demonstrates the complex regulatory mechanisms that balance pro-inflammatory and anti-inflammatory signals, highlighting the intricate interplay crucial for maintaining immune homeostasis. The specific role of CD109 in this crosstalk remains unclear (**Figure 1**). Given CD109’s emerging importance in regulating inflammation and its interaction with these pathways, exploring its precise involvement in TGF-β and NF-κB signaling network could offer valuable insights. It is important to understand whether CD109 exerts its influence on the NF-κB signaling network primarily at the receptor level—by modulating TLRs or other receptors— or through downstream signaling components, such as TAK1, TBK1, IKK complex, IκB degradation, and TAB2/3. Moreover, understanding the contexts in which CD109 either amplifies or suppresses NF-κB activity could shed light on its dual role in balancing immune responses. This could be particularly relevant in pathological conditions like fibrosis and cancer, where dysregulation of these pathways contributes to disease progression. Unraveling the exact points of interaction will not only clarify CD109’s function in immune homeostasis but may also reveal novel therapeutic targets for modulating inflammation and fibrosis. Additionally, examining how CD109 influences NF-κB-driven pro-inflammatory gene expression in different cell types, and how it may contribute to tissue-specific responses, could provide a more nuanced understanding of its regulatory role across various disease contexts.

## 3 CD109’s role in immune and inflammatory responses in tissues

**Figure 2:**
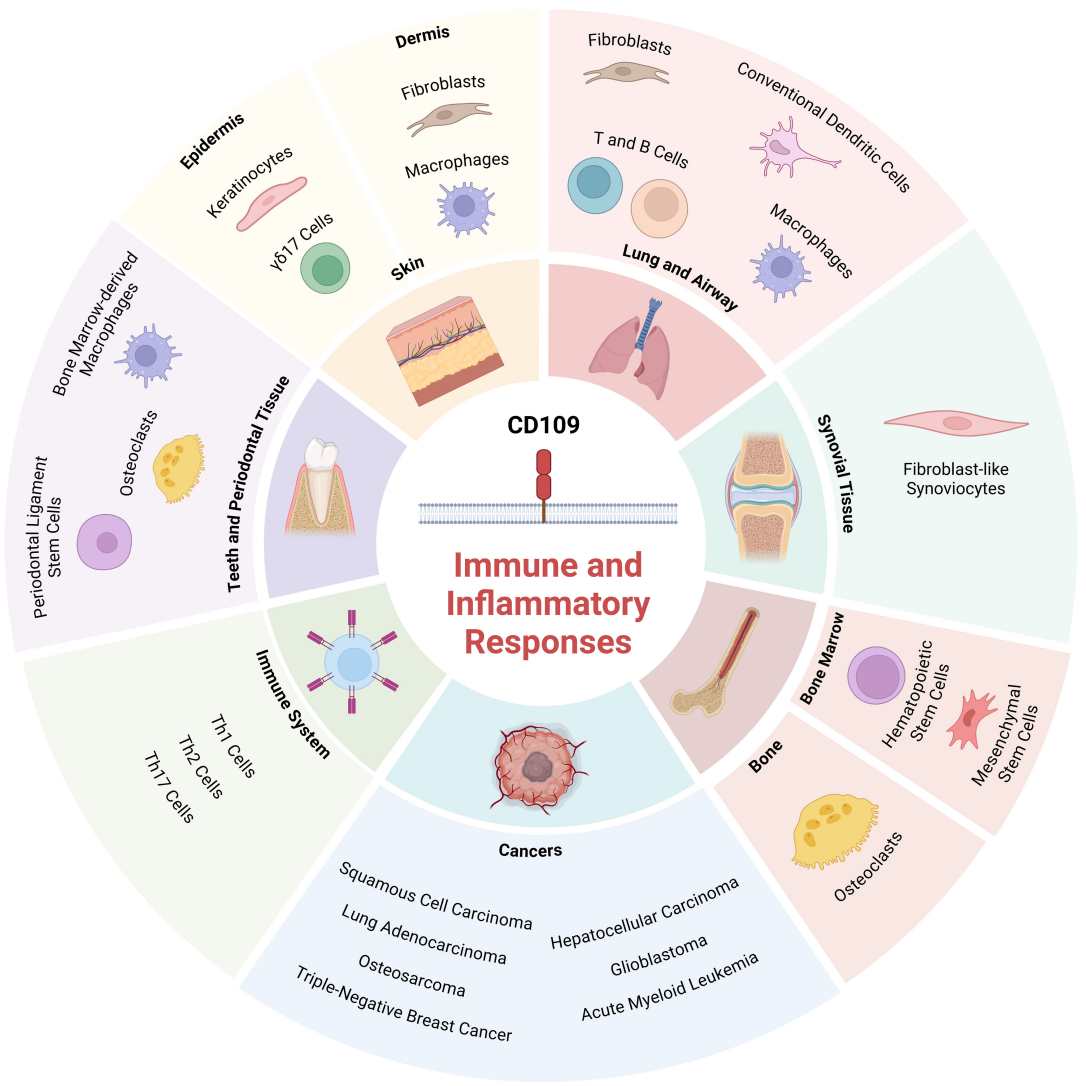
Role of CD109 in regulating immune and inflammatory responses in various tissues and cell types: The schematic diagram depicts tissues and cell types where CD109 is known to regulate immune and inflammatory responses. *Created with* *BioRender.com* .

CD109 plays a critical role in the regulation of immune and inflammatory responses across various tissues, cell types, and disease conditions. These include skin, lungs, synovial tissue, bone, bone marrow, peridontal tissue, as well T helper cells, fibrosis and cancers (**Table 1**; **Figure 2**). Emerging research has highlighted its multifaceted functions, particularly in modulating signaling pathways that govern inflammation and immunity.

**Table 1:** CD109’s role in immune and inflammatory responses in tissues and cell types.

Tissue	Cell Type	Main Finding	Reference
Skin (Epidermis)	Keratinocytes	CD109 overexpression decreases inflammation.	(11)
		CD109 overexpression inhibits immune cell recruitment during wound healing.	(11)
		CD109 overexpression decreases the expression of pro-inflammatory cytokines.	(11)
		CD109 deficiency leads to epidermal hyperplasia, impaired hair growth, and heightened immune cell infiltration.	(15)
		CD109 expression is reduced in psoriatic epidermis, while increased release from keratinocytes may drive molecular changes in psoriasis.	(28)
		CD109 is overexpressed in scleroderma and decreases fibrotic responses associated with the disease.	(29)
		The reduced expression of CD109 leads to diminished TGF-β signaling, leading to psoriasis by allowing increased Smad7 activity and subsequent inflammation.	(68)
	γδ17 cells	CD109 deletion in mice led to spontaneous skin inflammation (epidermal hyperplasia, increased cell proliferation, and the infiltration of neutrophils).	(27)
		CD109 is an important negative regulator of interactions between host and microbiota, limiting the activation of the cutaneous IL-23/IL-17 immune axis.	(27)
Skin (Dermis)	–	CD109 deficiency enhanced activation of the TGF-β/Smad pathway, induced macrophage infiltration, and increased levels of TNF-α and GM-CSF.	(69)
Lungs and Airway	Conventional Dendritic Cells (cDCs)	CD109 is mainly expressed in conventional dendritic cells (cDCs).	(70)
		CD109 expression is increased in lung conventional DC2s (cDC2s), but not cDC1s, following an allergic challenge.	(71)
		cDC2s deficient in CD109 displayed impaired cytokine production and high RUNX3, which suppresses Th2 cell differentiation.	(71)
	Lung Fibroblasts	In CD109-transgenic mice, CD109 is elevated in lung fibroblasts, T cells, B cells, and macrophages compared to wild-type mice.	(70)
	T cells		
	B cells		
	Macrophages		
Synovial Tissue	Fibroblast-Like Synoviocytes (FLSs)	CD109 knockdown led to a marked decrease in the phosphorylation of key signaling proteins, including Akt, NF-κB, STAT3, and p38 MAPK, in response to IL-1β and TNF-α stimulation.	(72)
		CD109 facilitates the activation and recruitment of leukocytes by promoting the production of CXCL-9 and CXCL-10 in rheumatoid arthritis FLSs.	(72)
		CD109 interacts with enolase 1 (ENO1), which activates p38 MAPK and NF-κB pathways, thus leading to elevated levels of pro-inflammatory mediators involved in rheumatoid arthritis.	(72)
Bone	Osteoclasts	CD109 expression significantly increases during RANKL-induced osteoclastogenesis.	(73)
		CD109 knockdown reduced the fusion of osteoclast precursors and the maturation of multinucleated osteoclasts.	(73)
Bone Marrow	Hematopoietic Stem Cells (HSCs)	CD109 regulates primitive HSCs, being most abundantly expressed in these early-stage cells and decreasing as they differentiate.	(74)
	Mesenchymal Stem Cells (MSCs)	CD109 secretion by human bone marrow mesenchymal stem cells lessens EMT and diminishes the stem cell-like properties of squamous cell carcinoma induced by TGF-β.	(75)
Teeth and Periodontal Tissue	Bone-Marrow-Derived Macrophage (BMDMs)	CD109 regulates BMDMs polarization towards an M1 phenotype in periodontal tissue.	(76)
	Periodontal Ligament Stem Cells (PDLSCs)	CD109 silencing reduces the expression of IL-6 and IL-1β, key inflammatory mediators in M1 macrophages.	(76)
		CD109 released by PDLSCs may reduce local immune-inflammatory responses.	(76)
	Osteoclasts	CD109 promotes osteoclast formation and activity induced by PDLSCs, leading to increased osteoclast numbers and pro-inflammatory factor secretion under mechanical force stimulation.	(76)
		*In vivo* CD109 inhibition reduces osteoclast formation, suppresses inflammation, and improves bone density in periodontal tissues.	(76)
Immune System	T Helper Cells	CD109 expression is linked to reduced TGF-β signaling in Th2 cells, impacting their differentiation and function in allergic diseases such as chronic rhinosinusitis with nasal polyps	(77)
		CD109 is an important molecular switch on CD4+ T cells, regulating the balance between Th1 and Th17 pathways.	(78)
Cancers	Squamous Cell Carcinoma (SCC)	CD109 is essential for tumor progression by regulating EGFR/Akt signaling, usually involved in inflammation-related pathways.	(79)
		CD109 induces EGFR-mediated STAT3 phosphorylation, which supports SCC cell migration, proliferation, and the cancer stem cell phenotype, highlighting its role in enhancing tumor aggressiveness and inflammation.	(80)
		CD109’s secretion by human bone marrow mesenchymal stem cells reduces EMT and diminishes the stem cell-like properties of squamous cell carcinoma induced by TGF-β.	(75)
	Lung Adenocarcinoma	CD109 acts as a pro-metastatic factor in lung adenocarcinoma by activating the JAK/STAT3 signaling pathway, which is also integral to inflammation and immune responses.	(81)
		CD109 interacts with LTBP1.	(82)
		Increased CD109 expression was found to enhance stromal TGF-β activation in the presence of LTBP1.	(82)
		CD109 interacts with EGFR to activate the Akt/mTOR pathway, driving tumor growth.	(83)
	Osteosarcoma	CD109 overexpression correlates with poor prognosis and promotes tumor cell migration via suppression of BMP-2-induced Smad1/5/9 phosphorylation.	(84)
	Hepatocellular Carcinoma	Reduced CD109 expression on tumor-associated endothelial cells (TEC) is linked to advanced tumor features, including larger size, microvascular invasion, and increased tumor progression, proliferation, migration, and hepatoma cell invasion via enhanced IL-8 secretion.	(116)
	Sk-MG-1 Glioblastoma	CD109 negatively regulates TGF-β1 signaling and enhances EGF signaling.	(85)
	Glioma Stem Cells (GSCs)	CD109 interacts with GP130 to activate the IL-6/STAT3 pathway, essential for inflammation and immune cell recruitment.	(86)
	Triple-Negative Breast Cancer (TNBC)	CD109 is highly expressed in TNBC, correlating with chemotherapeutic resistance and poor outcomes, and may influence tumor progression and inflammation, highlighting the need for further research.	(87, 88)
	Acute Myeloid Leukemia (AML)	CD109 is overexpressed in AML cell lines compared to normal bone marrow stromal cells, highlighting it as a potential biomarker for AML.	(89)
		CD109 is linked to the deregulation of TCR signaling, a key pathway for immune responses.	(89)

### 3.1 Skin

CD109 is increasingly recognized for its significant role in modulating inflammatory responses in the skin, particularly in keratinocytes within the epidermis. We have shown that CD109 overexpression in the epidermis of mice decreases inflammation and reduces the number of neutrophils and macrophages during wound healing compared to their wild-type littermates (11). This suggests that epidermal these inhibits immune cell recruitment during wound healing (11). In addition, these transgenic (TG) mice display a reduction in the mRNA expression of pro-inflammatory cytokines, IL-1α and MCP-1 (11). Given that these proinflammatory cytokines are chemotactic for neutrophils and macrophages, the reduction in their levels likely contributes to the decreased immune cell recruitment to the wound site observed in CD109 TG mice. The decreased TGF-β signaling through the Smad2/3 pathway during wound healing in CD109 TG mice likely explain the reduced expression of the proinflammatory cytokines IL-1α and MCP-1 (35, 36), despite elevated TGF-β levels during the inflammatory phase of wound healing (11).

Psoriasis is an autoimmune inflammatory skin disease where the immune system erroneously attacks healthy skin cells, leading to inflammation and accelerated epidermal turnover (90). Dysregulated TGF-β signaling drives keratinocyte hyperproliferation, promotes Th17 cell-mediated inflammation, and enhances macrophage cytokine release, perpetuating the disease (91). Analysis of CD109 expression in psoriasis patients by our group revealed that CD109 protein expression is markedly decreased in psoriatic epidermis as compared to adjacent uninvolved skin (28). In contrast, CD109 mRNA expression is unchanged in psoriatic plaques in comparison with normal skin. This raises the possibility that CD109 protein release is enhanced in psoriatic keratinocytes and suggest that aberrant CD109 release from the cell surface in human keratinocytes may induce molecular changes that are observed in psoriasis (28). A recent report confirmed this finding and demonstrated that decreased CD109 expression correlates with increased levels of Smad7 which may lead to enhanced inflammation and cell proliferation (68). Another group has highlighted the role of CD109 as a key regulator of the skin’s IL-23/IL-17 immune axis and γδ17 cells, a subset of T cells characterized by their production of the pro-inflammatory cytokine IL-17 (27). In this study, deletion of CD109 in mice led to spontaneous skin inflammation, characterized by epidermal hyperplasia, increased cell proliferation, and the infiltration of neutrophils into both the epidermal and dermal layers of the skin. The epidermal and dermal layers of the ears of CD109 knockout mice revealed a significant increase in cytokine gene expression associated with the IL-23/IL-17 immune axis as compared to wild-type controls. This dysregulation in the IL-23/IL-17 pathway also led to heightened psoriasiform inflammation following imiquimod treatment. Moreover, CD109 acts in a skin-specific and cell-extrinsic manner to control IL-23-dependent γδ17 cell activation induced by commensal microbiota. Overall, CD109 is an important negative regulator of interactions between host and microbiota, limiting the activation of the cutaneous IL-23/IL-17 immune axis (27). This is consistent with the previous report by Mii et al. which demonstrated that CD109 deficiency leads to epidermal hyperplasia and abnormalities in skin appendages, including hair follicles (15). These mice show increased sebum accumulation and inflammatory cell infiltration, including T cells, neutrophils, and a small number of B cells, in the dermis. Keratinocytes from these mice show an increase in STAT3 phosphorylation, associated with increased proliferation, impaired differentiation, and psoriasis-like skin changes with inflammatory infiltrates *in vivo* (15). Together, these findings suggest that inflammation can drive epidermal hyperplasia by promoting keratinocyte proliferation and disrupting normal skin function.

Scleroderma is an autoimmune disease that causes inflammation and fibrosis primarily affecting skin and internal organs such as lungs and kidneys (29). As previously demonstrated by us, CD109 expression in scleroderma is significantly elevated in skin tissue and fibroblasts compared to healthy controls, likely as an adaptive response (29). A link between CD109 expression and inflammation in scleroderma remains to be established. While CD109's essential role in the epidermis was established through findings from its critical function in psoriasis, it also plays a key role in the dermis. The dermis, which contains a rich matrix of connective tissue and various cell types including immune cells like macrophages and mast cells, as well as structural cells like fibroblasts, represents another important context where CD109 could influence inflammatory responses. The dermal cells are crucial for tissue repair and immune responses, and studies of CD109 function in these dermal cells could provide insights into how this protein regulates inflammation beyond the epidermal layer. We have previously shown that overexpression of CD109 specifically in the epidermis regulates dermal function and that CD109 may provide a critical link in promoting dermal-epidermal interaction (26). In these mice, CD109 overexpression leads to decreased inflammatory responses including macrophage and neutrophil recruitment, granulation tissue formation during wound healing, when compared to wild-type littermates, as mentioned above (11), and this is associated with decreased TGF-β/Smad2/3 signaling (11) and NF-κB pathway inhibition (12, 13) as well as decrease in fibrotic parameters (14). Consistent with this CD109 global knockout mice display opposite effects with heightened immune cell infiltration, as well as epidermal hyperplasia, and impaired hair growth (15) while exhibiting enhanced TGF-β/Smad2/3 signaling and increased skin fibrosis (16). Recent findings from inflammatory tumorigenesis experiments in CD109-deficient mice provide evidence of CD109’s role in modulating dermal immune and inflammatory responses (69). In CD109-deficient mice, enhanced activation of the TGF-β/Smad pathway in the dermis was associated with increased macrophage infiltration and elevated levels of inflammatory cytokines such as TNF-α and GM-CSF (69). These findings suggest that CD109’s absence amplifies dermal TGF-β signaling and alters the immune cell dynamics within the dermal layer, potentially contributing to a tumor-suppressive microenvironment in early carcinogenesis. The observed interplay between CD109, TGF-β signaling, and immune cell activity in the dermis highlights its potential significance in regulating inflammation and tissue homeostasis beyond the epidermis, warranting further investigation into its dermal-specific functions in inflammatory skin diseases.

### 3.2 Lungs and airways

Idiopathic pulmonary fibrosis (IPF) is one of the pulmonary diseases in which CD109 expression is dysregulated (70). However, its role in this condition’s immune and inflammatory responses has not yet been thoroughly investigated. Various cell types are involved in the pathophysiology of pulmonary fibrosis, with inflammatory cells such as macrophages, T cells, and B cells playing a crucial role (92). In the lungs of patients with IPF, CD109 is prominently expressed in basal cells, as well as in endothelial cells and macrophages although, under normal conditions, CD109 is mainly expressed in conventional dendritic cells (cDCs) (Lung cell Atlas (https://asthma.cellgeni.sanger.ac.uk), and IPF Cell Atlas (http://www.ipfcellatlas.com)). Bleomycin treatment leads to enhanced CD109 levels in lung fibroblasts and most immune cells while CD109 levels are decreased in the cDC1 subtype in mice (70). After bleomycin treatment, a higher accumulation of inflammatory cells and greater collagen deposition was observed in the lungs of wild-type mice compared to CD109 TG mice (70). These findings suggest that CD109 plays a role in the inflammatory aspect of pulmonary fibrosis.

Macrophage polarization is a key factor in the development of IPF (93). M1 macrophages, which are pro-inflammatory, contribute to early lung damage and fibrosis, while M2 macrophages, which are anti-inflammatory, aid in tissue repair and fibrosis (93). Although CD109’s role in macrophage polarization has not yet been investigated, exploring this could provide valuable insights and potential therapeutic opportunities.

CD109 has also been shown to be associated with inflammatory processes related to the airway and bronchial asthma (71). Asthma is a chronic airway inflammatory disease marked by airway hyperreactivity (AHR) and eosinophilic inflammation (94). Dendritic cells (DCs) are crucial in asthma development by presenting allergens, which drive T-helper cell type 2 (Th2) responses and eosinophil inflammation (94). Mice deficient in CD109 and sensitized to allergens showed diminished airway hyperreactivity (AHR), eosinophilic inflammation, and reduced levels of Th2 cytokines compared to their wild-type counterparts (71). CD109 expression is also increased in lung conventional DC2s (cDC2s), but not cDC1s, following an allergic challenge (71). cDC2s deficient in CD109 displayed impaired cytokine production and high RUNX3 (Runt-related transcription factor 3) expression, suppressing Th2 cell differentiation (71). RUNX3, a critical transcription factor, cooperates with TGF-β intracellular signal transducers Smad2 and Smad3, forming complexes that regulate TGF-β target genes (95). This interaction highlights the connection between CD109, TGF-β signaling, and allergic responses, as CD109 deficiency impacts both RUNX3 activity and downstream effects on Th2-mediated inflammation. Additionally, the adoptive transfer of bone marrow-derived CD109^-/-^ dendritic cells loaded with allergens did not induce AHR or eosinophilic inflammation in WT mice, reinforcing the role of CD109 in these processes (71). Consistent with this, when administered with anti-CD109 monoclonal antibodies, mice exhibited a significant reduction in AHR and eosinophilic inflammation during both allergen sensitization and allergen challenge (71).

CD109 significantly influences inflammation in pulmonary tissues, suggesting that it affects both IPF and asthma. Investigating its specific role in these and other immune processes in the lung could reveal new therapeutic targets and deepen our understanding of its broader impact on immune regulation and inflammation in lung diseases.

### 3.3 Bone and related tissues

#### 3.3.1 Bone

CD109 has been implicated in inflammatory processes within bone tissue, suggesting a potential role in regulating both immune responses and bone remodeling (73, 96). Osteoclasts, which are large multinucleated cells derived from the monocyte/macrophage lineage, are broadly considered inflammatory cells due to their involvement in bone resorption and their response to inflammatory mediators (97). It was found that CD109 expression is significantly increased during RANKL-induced osteoclastogenesis, and knockdown of CD109 resulted in reduced fusion of osteoclast precursors and fewer mature multinucleated osteoclasts (73). These findings suggest that CD109 is a key regulator of osteoclast differentiation and may play an important role in inflammatory bone remodeling. Additional *in vivo* studies revealed that CD109 deficiency induces an osteoporosis-like phenotype characterized by reduced bone volume and increased bone turnover (96).

#### 3.3.2 Bone marrow

The bone marrow is crucial for the production and development of blood cells, including immune cells essential for both innate and adaptive immunity (98), in addition to regulating their function through cytokines and growth factors (98). Hematopoietic stem cells (HSCs) in the bone marrow produce all blood cells, including those essential for immune responses and regulation of inflammation (99).

CD109 is most abundantly expressed in primitive HSCs with its expression decreasing as they differentiate (74). In immune-mediated bone marrow failure, inflammation damages the bone marrow by targeting its cells and disrupting blood and immune cell production (100). CD109 suppresses TGF-β signaling in HSCs, and the lack of CD109 may increase their sensitivity to TGF-β, thus leading to preferential commitment of erythroid progenitor cells to mature red blood cells in immune-mediated bone marrow failure (74), suggesting that CD109 by modulating TGF-β signaling, regulates this inflammatory response in the bone marrow.

We have previously shown that CD109 released into the secretome of bone marrow is able to inhibit TGF-β epithelial to mesenchymal transition (EMT) and stemness of SCC cells (75). While the process of EMT has been implicated in hematopoietic malignancies (101), whether CD109-medited regulation of EMT and inflammation plays a role in the progression of the malignant progression of these cancers remains to be determined. EMT and inflammation are intricately connected in cancer (102). Inflammatory cytokines like TNF-α and IL-6, along with chemokines such as CXCL-8, drive EMT by activating transcription factors such as Snail, Twist, and Zeb (102). NF-κB, a key regulator in this process, is activated by these cytokines and plays a critical role in promoting EMT (102). The NF-κB pathway may be considered as a connecting link between inflammation and tumorigenesis through activation of antiapoptotic genes, angiogenesis factors, and proinflammatory cytokines (102). Delineation of the mechanism by which CD109 may regulate EMT and inflammation in hematopoietic malignancies will provide key insights and may lead to avenues for therapeutic intervention.

#### 3.3.3 Synovial tissue

Rheumatoid arthritis (RA) is a chronic autoimmune disease characterized by persistent inflammation of the synovial joints and hyperplasia of fibroblast-like synoviocytes (FLSs) (103). CD109 is highly expressed in the synovial tissues of individuals with RA (72). CD109 modulates RA FLS-mediated inflammation independently of TGF-β signaling by interacting with enolase 1 (ENO1) (72). ENO1 activates intracellular p38 MAPK and NF-κB pathways, which leads to elevated levels of pro-inflammatory mediators involved in RA, such as IL-1β, IL-6, and TNF-α (104).

CD109 is essential for the increased production of key inflammatory cytokines implicated in RA FLS-mediated inflammation, such as IL-6, IL-8, MMP-1, and MMP-3 (72). RNA interference studies have shown that silencing CD109 significantly lowers these cytokine levels (72). CD109 knockdown led to a marked decrease in the phosphorylation of key signaling proteins, including Akt, NF-κB, STAT3, and p38 MAPK, in response to IL-1β and TNF-α stimulation (72). Additionally, CD109 facilitates the activation and recruitment of leukocytes by promoting the production of CXCL-9 and CXCL-10 in RA FLSs (72). CD109 deficiency resulted in decreased Receptor Activator of NF-κB Ligand (RANKL) expression in FLSs, which is crucial for inflammation through its role in osteoclast activation and bone resorption (72, 105). Overall, CD109 is critical for cytokine production, inflammatory signaling, and immune cell activation in RA (72). 

#### 3.3.4 Teeth and periodontal tissue

Macrophage polarization is essential in inflammation because it determines the macrophages’ role in either promoting inflammation and fighting pathogens (M1) or resolving inflammation and aiding in tissue repair (M2) (106). CD109 seems to regulate that process in periodontal tissue. CD109 expression on force-treated periodontal ligament stem cells (PDLSCs) promotes bone-marrow-derived macrophages (BMDMs) to polarize toward the M1 phenotype and reduces their polarization to the M2 phenotype (76). Moreover, silencing CD109 in the periodontal ligament reduces the expression of IL-6 and IL-1β, key inflammatory mediators in M1 macrophages (76). CD109 released by PDLSCs may reduce local immune-inflammatory responses (76). This suggests CD109’s role in PDLSCs’ immunomodulation and highlights its secretion via extracellular vesicles as a novel mechanism for regulating the immune microenvironment (76, 107). Furthermore, CD109 promotes osteoclast formation and activity induced by PDLSCs, leading to increased osteoclast numbers and pro-inflammatory factor secretion under mechanical force stimulation (76). In contrast, inhibiting CD109 *in vivo* reduces osteoclast formation, suppresses inflammation, and improves bone density in periodontal tissues (76).

The precise signaling pathway by which CD109 modulates these immune responses remains to be determined in periodontal tissues. However, considering NF-κB’s established role in M1/M2 polarization (51), it is plausible that CD109 might exert its effects through the NF-κB pathway. Elucidation of the mechanisms by which CD109 influences macrophage polarization and broader immune-inflammatory processes may offer new insights into the therapeutic targeting of CD109 for controlling inflammation and immune responses in periodontal tissues.

### 3.4 Immune system

CD109 likely plays a key role in maintaining immune homeostasis and mitigating inflammatory diseases by regulating specific immune cell functions. For example, CD109 induces the differentiation and function of T helper cells. In nasal polyps, a distinct subset of Th2 cells expressing CD109 was identified (77). These cells produce IL-10, a cytokine with immunosuppressive functions, distinguishing them from classical ‘chemoattractant receptor-homologous molecule expressed on Th2 cells’ (CRTH2) Th2 cells (77). CD109 is not widely expressed in other Th cell subsets and may serve as a marker for regulatory Th2 cells (77). In addition, CD109 expression is linked to reduced TGF-β signaling in Th2 cells, impacting their differentiation and function in allergic diseases such as chronic rhinosinusitis with nasal polyps (77).

CD109’s role in Th1 and Th17 pathways has been well documented in ongoing investigations (78). CD109 seems to be a direct target of CD46 in Th1 cells, a regulator of T cell-induced inflammation (78). Knockout or disruption of CD109 in CD4+ T cells lead to uncontrolled Th1 and Th17 activation, cytokine secretion (IL-10 and IL-17), severe inflammatory disease, and exaggerated STAT3 activation, highlighting its role in maintaining immune balance through SOCS3-mediated inhibition of STAT3 activity (78). Thus, CD109’s regulation of TGF-β signaling and its impact on cytokine production (like IL-10 and IL-17) appear to be crucial for balancing immune responses and controlling inflammation. By modulating Th1, Th2, and Th17 pathways, CD109 likely help prevent excessive inflammation and autoimmunity, and thus playing a crucial role in maintaining immune balance and reducing the risk of inflammatory diseases.

## 4 CD109’s role in various cancers

### 4.1 Squamous cell carcinoma

In squamous cell carcinoma (SCC), CD109 is essential for tumor progression and is associated with increased EGFR expression/stabilization, and enhanced EGFR/Akt signaling, which are crucial for maintaining epithelial morphology and cellular stemness, as shown by us (79) and others (80). This interaction suggests a link to inflammation-related pathways, as EGFR signaling is often involved in cancer-related inflammatory responses (108). Furthermore, CD109 induces EGFR-mediated STAT3 phosphorylation, which supports SCC cell migration, proliferation, and the cancer stem cell phenotype *in vitro*, suggesting its role in enhancing tumor aggressiveness and inflammation (80). In addition, our findings have shown that CD109 acts as a gatekeeper of the epithelial trait by suppressing epithelial to mesenchymal transition (EMT) in SCC cells (109), and that CD109 present in the secretome of human bone marrow mesenchymal stem cells is at least partially responsible for the secretome’s effect in decreasing EMT (75). A possible mechanistic explanation for the paradox that CD109 loss promotes EMT, but forfeits tumorigenicity and metastatic ability in SCC cells is that the loss of CD109 action leads to an irreversible and tumor-suppressive EMT program which generates fully differentiated mesenchymal phenotype (110). Emerging evidence suggests that it is the hybrid epithelial/mesenchymal (E/M) cells, rather than the cells that have undergone complete EMT, that are involved in cancer cell migration and invasion, resulting in metastasis (110, 111). However, it is important to note that EMT is a reversible process regulated by multiple factors including master transcription factors and metabolic states (110, 111), and thus the role of CD109 in the EMT process is likely to be context-dependent. Given the interplay between EMT and inflammation in cancer, where pro-inflammatory cytokines like TGF-β and NF-κB drive EMT (68), CD109’s capacity to modulate these processes and cancer progression suggests that CD109 promotes SCC tumor progression through these pathways.

### 4.2 Lung adenocarcinoma

CD109 is highly expressed in lung adenocarcinoma, where it promotes tumor progression, metastasis, and is associated with poor patient outcomes (83). CD109 acts as a pro-metastatic factor in lung adenocarcinoma by activating the JAK/STAT3 signaling pathway, which is also integral to inflammation and immune responses (81, 112). Its role in metastasis appears to be context-dependent, as suggested by its differential upregulation and function in metastatic cells compared to primary tumors, with JAK/STAT3 activation being more pronounced in the metastatic state (112). CD109's interactions with the tumor microenvironment may vary depending on the local signaling context, potentially involving both autocrine and paracrine mechanisms​​ (112).

Contrary to its typical role as a negative regulator of TGF-β signaling, CD109 may actually promote TGF-β action, as has been recently reported that CD109 is involved in the activation of latent TGF-β to active TGF-β, enhancing tumor invasion and inflammation. Latent TGF-β Binding Protein 1 (LTBP1) was identified as a CD109-interacting protein in lung adenocarcinoma and their interaction is crucial for tumor invasion and inflammation in lung adenocarcinoma (82). LTBP1’s role in TGF-β activation leading to enhanced TGF-β/Smad signaling and inflammation are well-documented in epilepsy models, where inhibiting LTBP1 expression has shown to have neuroprotective effects. It is possible that similar mechanisms may also be relevant in cancer (113).

In lung adenocarcinoma, EGFR mutations are prevalent and are often associated with increased levels of inflammatory cytokines like IL-6 and TNF-α, which contribute to tumor progression (114). CD109 may amplify this process, as it interacts with EGFR to activate the Akt/mTOR pathway, driving tumor growth (83). In this regard, inhibiting CD109 is known to reduce EGFR signaling, suppress the Akt/mTOR pathway, and increase tumor cell sensitivity to EGFR inhibitors, highlighting its potential as a promising therapeutic target in lung cancer (83).

### 4.3 Osteosarcoma

CD109 is highly expressed in osteosarcoma, promote tumor cell migration via suppression of bone morphogenetic protein-2 (BMP-2)-induced Smad1/5/9 phosphorylation, where its expression correlates with poor prognosis (84). BMPs are not only involved in bone development but also play roles in regulating inflammation, as they can modulate cytokine production and immune responses in the tumor microenvironment (115). While CD109’s role in TGF-β signaling appears limited in osteosarcoma, its involvement in BMP signaling highlights a distinct mechanism in this cancer (84). Given its established connections to inflammatory processes in bone and its modulation of BMP signaling, CD109’s role in inflammation within the osteosarcoma tumor microenvironment warrants further investigation. It is plausible that CD109 could influence BMP-mediated inflammatory responses, cytokine production, and immune cell infiltration, thereby contributing to the tumor’s inflammatory and metastatic potential. This hypothesis strengthens the argument for studying CD109 as a potential link between inflammation, bone remodeling, and cancer progression in osteosarcoma.

### 4.4 Hepatocellular carcinoma

In hepatocellular carcinoma, CD109 plays a pivotal role in the interaction between tumor-associated endothelial cells (TEC) and the tumor microenvironment, particularly in the context of inflammation and immune response (116). Reduced CD109 expression on TEC correlates with advanced tumor characteristics, such as larger size and increased microvascular invasion (116), and is also associated with enhanced tumor progression, proliferation, migration, and invasion of hepatoma cells through increased secretion of interleukin-8 (IL-8), a potent proinflammatory cytokine (116). The regulation of IL-8 involves the TGF-β/Akt/NF-κB signaling pathway, which is integral to managing inflammatory responses and enhancing tumor growth (116). Therefore, CD109’s impact on TEC not only affects tumor advancement and prognosis but also serves as a significant regulator of the local immune environment.

### 4.5 Glioblastoma

CD109 negatively regulates TGF-β1 signaling and enhances EGF signaling in glioblastoma cells, and might represent a critical link in the balance of signaling between these two oncogenic pathways that control inflammatory responses, cell migration and invasion (85). CD109 binds to the EGF Receptor (EGFR) in SK-MG-1 cells, but this binding is abolished upon EGF stimulation, highlighting the dynamic interaction between CD109 and EGFR (85). CD109 may regulate EGFR signaling and its associated responses, such as its dual role in inflammatory responses in endothelial cells, which has been shown to be context-dependent (108).

In glioma stem cells (known to drive the propagation and therapy resistance of glioblastomas), CD109 interacts with glycoprotein 130 (GP130) to activate the IL-6/STAT3 pathway (86). The IL-6/JAK2/STAT3 signaling pathway plays a crucial role in inflammation and is currently recognized as one of the key pathways in this process (81). Il-6 recruits immune cells to the tumor microenvironment, which in turn promotes the secretion of more pro-inflammatory cytokines (117). As a critical regulator of the GP130/IL-6/STAT3 pathway, CD109 may act as a crucial link between chronic inflammation and tumor development.

### 4.6 Triple-negative breast cancer

In triple-negative breast cancer (TNBC), CD109 expression is increased in cancer stem cells from TNBC patients as compared to non-TNBC samples, and is linked to chemotherapeutic resistance, increased distant metastasis, and poorer disease-specific survival (87). While CD109’s exact role in TNBC remains unclear, its association with breast cancer stem-like cells suggests a potential involvement in inflammation in TNBC leading to high mortality and tumor progression (88).

In TNBC, CD109 may modulate key inflammatory and tumor-promoting pathways, such as TGF-β, EGFR, and GP130, which are all implicated in TNBC progression (87, 118, 119). CD109’s known ability to regulate TGF-β signaling may influence the dynamic balance between TGF-β’s tumor-suppressive and pro-tumorigenic effects, particularly in the inflammatory tumor microenvironment. Additionally, CD109’s interaction with EGFR could amplify pro-inflammatory cytokine production, such as IL-6 and IL-8, by activating downstream pathways like Akt/mTOR and NF-κB, thereby enhancing tumor growth, immune suppression, and potentially contributing to chemoresistance. Furthermore, CD109’s potential involvement in the IL-6/GP130/STAT3 axis, a key driver of chronic inflammation and cancer stem cell survival, suggests it may help sustain an inflammatory environment that fosters metastasis and immune evasion (120, 121). By integrating signals from these pathways, CD109 could act as a central mediator that perpetuates inflammation and immune modulation, highlighting its importance in TNBC progression and the urgent need for further investigation into its mechanisms.

### 4.7 Acute myeloid leukemia

CD109 has been studied predominantly in the context of solid tumors, but its role in hematological neoplasms is gaining attention. CD109 has emerged as a potential biomarker for Acute Myeloid Leukemia (AML), playing crucial roles in immune attention and inflammation (89). It is significantly overexpressed in AML cell lines compared to normal bone marrow stromal cells, and this upregulation correlates with poor patient survival, suggesting its value as a diagnostic and prognostic marker (89). CD109 is linked to the deregulation of TCR signaling, a key determinant of T cell-mediated immune responses (89). This includes reduced expression of co-stimulatory immune checkpoint molecules like CD28 and CD226, which impairs T-cell activation and immune surveillance (89). This deregulation, combined with the competitive binding of inhibitory checkpoint molecules such as CTLA-4, leads to a diminished immune response, allowing AML cells to evade detection and destruction (89).

Beyond AML, CD109 has the potential to play similar roles in other hematological malignancies. For example, its ability to modulate TGF-β non-canonical pathways, such as JAK-STAT3 and NF-κB, suggests a broader impact on the inflammatory and immune characteristics of diseases like lymphomas (122). In these conditions, CD109 could contribute to immune dysfunction by promoting a suppressive tumor microenvironment, characterized by impaired cytotoxic lymphocyte activity, cytokine imbalances, and chronic inflammation.

Hematological neoplasms frequently exhibit inflammatory tumor microenvironments due to dysregulated cytokine production, immune checkpoint disruption, and the recruitment of suppressive immune cells such as regulatory T cells and myeloid-derived suppressor cells (MDSCs) (123, 124). CD109 may exacerbate these conditions by modulating the TGF-β pathway. For instance, the loss of TGF-β control has been linked to the persistence of pro-inflammatory cytokines such as IL-6 and TNF-α, which are hallmarks of the inflammatory milieu in hematological malignancies (125, 126). This inflammatory environment not only supports tumor progression but also contributes to resistance against immune-based therapies.

The identification of CD109 as a biomarker in AML suggests its potential utility in other blood cancers, both as a diagnostic tool and a therapeutic target. By influencing immune regulation and inflammation, CD109 could represent a key hub in the interaction between hematological neoplasms and their inflammatory microenvironments. Its role in shaping these environments provides a rationale for further studies to elucidate its functions in other blood cancers, such as lymphomas and myelomas, where inflammation is similarly pivotal. Understanding CD109’s contributions to these processes could pave the way for novel therapeutic strategies aimed at targeting inflammation and immune dysregulation in hematological malignancies.

## 5 Concluding remarks

CD109 is a multifaceted regulator of immune and inflammatory responses across various tissues and conditions. In the skin, CD109 modulates inflammation by influencing cytokine levels and immune cell recruitment, impacting conditions such as wound healing, psoriasis, and scleroderma. In the lungs, CD109 affects inflammation and fibrosis, potentially through its interaction with immune cells and signaling like that of TGF-β. In synovial tissue, CD109 drives inflammation in rheumatoid arthritis by regulating cytokine production and signaling. It also plays a critical role in the bone marrow, affecting hematopoietic stem cell differentiation and cell behavior, which influences inflammation, bone marrow homeostasis, and cancer progression. In the bone, CD109 plays a dual role by modulating osteoclast formation and inflammatory responses, while influencing bone remodeling and density through its regulation of immune regulatory signaling pathways. CD109 may play a key role in immune balance by preventing excessive inflammation and autoimmunity via its regulation of T helper cells (Th1, Th2, and Th17 pathways). CD109's involvement in cancers such as lung adenocarcinoma, glioblastomas, hepatocellular carcinomas, breast cancer, leukemia, and squamous cell carcinomas highlights its significant impact on immune responses and oncogenic process. Further research is needed to elucidate the specific mechanisms by which CD109 operates in these contexts.

CD109 influences key inflammatory pathways, including TGF-β/NF-κB, EGF/STAT3, and various inflammatory cytokines like IL-6 and IL-8, impacting inflammatory processes such as immune cell recruitment, macrophage polarization, immune microenvironment, EMT, and tumor progression. Future research could focus on elucidating the precise mechanisms by which CD109 regulates the inflammatory pathways to maintain tissue homeostasis in health and promote oncogenic progression in cancer.
